# Home‐Based High‐Intensity Interval Training for People With Parkinson's: A Randomized, Controlled, Feasibility Trial

**DOI:** 10.1002/hsr2.71024

**Published:** 2025-07-14

**Authors:** Conrad Harpham, Hilary Gunn, Jonathan Marsden, Raul Bescos‐Garcia, Christopher Murgatroyd, Luke Connolly

**Affiliations:** ^1^ University of Plymouth Plymouth UK; ^2^ Manchester Metropolitan University Manchester UK

**Keywords:** exercise, HIIT, home‐based, Parkinson's disease, rehabilitation

## Abstract

**Background and Aims:**

Home‐based high‐intensity interval training (HIIT) could be feasible and useful for people with Parkinson's (PwP). However, no home‐based HIIT program for PwP has been undertaken. This trial was designed to obtain preliminary data regarding the feasibility, acceptability and safety of HIIT‐Home4Parkinson's (HH4P), a previously co‐created home‐based HIIT program for PwP, explore outcomes that may be sensitive to change, and inform the implementation of a potential full trial.

**Methods:**

A randomized, controlled feasibility trial was undertaken. Thirteen independently mobile PwP of Hoehn and Yahr stages 1–3 were randomized to the 12‐week, three times weekly HH4P HIIT program (*n* = 7), or usual care (*n* = 6). Feasibility and safety outcomes included aspects such as program completion, adherence, exercise intensity and adverse effects and events. Potential primary outcomes for a full trial were serum brain‐derived neurotrophic factor, maximal oxygen uptake and the Unified Parkinson's Disease Rating Scale part III. Process evaluation with a qualitative aspect explored implementation fidelity and participant thoughts and feelings.

**Results:**

Six HIIT participants completed the program, with one withdrawing due to an unrelated back injury. Mean exercise adherence was 78.4%, while the mean exercise intensity was 77.2% HR_max_ per session, with three participants not achieving mean 75% HR_max_. HIIT related adverse effects were minor and temporary, and the majority of exercise program and delivery procedures were deemed feasible and acceptable by participants. When compared to controls, the HIIT group did not experience benefits in any of the potential primary outcomes.

**Conclusion:**

Preliminary data suggests that home‐based HIIT could be feasible, safe and acceptable for some PwP, although the capacity to stimulate the required exercise intensity, along with the potential benefits remain uncertain. Progression to a full HH4P trial cannot be recommended until further evaluation of aspects such as exercise type and model of support is undertaken.

**Trial Registration:**

ClinicalTrials.gov **NCT05485428**.

## 1 Background and Aims

Parkinson's disease (Parkinson's) is a common neurodegenerative condition characterized by bradykinesia, tremor, stiffness, and postural instability along with a range of nonmotor features [1]. PwP can also demonstrate reduced cardiovascular fitness compared to controls [2], potentially leading to additional health and wellbeing complications. High‐intensity interval training (HIIT) has been found to be feasible, safe and acceptable for some PwP, and can improve motor symptoms, maximal oxygen uptake (VO_2max_) and levels of brain‐derived neurotrophic factor (BDNF), thought to have neuroprotective properties [3, 4, 5]. However, extended adherence to HIIT programs could be problematic for PwP [3], who experience barriers to exercise such as perceived lack of time, expense, and travel limitations due to motor symptoms [6, 7]. Finding ways to overcome these barriers is therefore a priority. Home‐based exercise programs offer putative logistical benefits, and home‐based balance, gait, and aerobic exercise programs have been found to be feasible and beneficial for PwP [8, 9]. However as yet, no home‐based HIIT program for PwP has been undertaken. HIIT‐Home4Parkinson's (HH4P) is a novel, 12‐week home‐based HIIT program for PwP, based on adaptability, individualization and remote supervision. The HH4P program was previously co‐created by researchers, clinicians, PwP and family members within an iterative process of focus groups, exercise testing and interviews [10]. Having optimally developed the HH4P protocol [11], it was vital to undertake a feasibility trial to explore the practicality and potential benefits of the program. Therefore, the HH4P randomized, controlled feasibility trial was undertaken to obtain preliminary data regarding the feasibility, acceptability, safety and utility of HH4P. The trial had three key aims with associated objectives [11];

A. To evaluate the feasibility, safety and acceptability of the HH4P 12‐week program, by determining; 1, program safety, 2, program adherence, 3, program completion, 4, achieved exercise intensity, 5, participant acceptance of the program and delivery procedures, 6, practicality of intervention resources, and 7, intervention fidelity [7].

B. To identify the clinical and physiological outcomes that could be feasible and sensitive to change compared to usual care in a full home‐based HIIT trial for PwP, by determining; 1, responsiveness to change in mechanistic, physiological, and clinical outcomes to inform the selection of primary and secondary outcomes for a definitive trial, and 2, feasibility of potential primary and secondary outcome measure procedures, including rates of completion.

C. To elucidate the key methodological considerations for the implementation of a full home‐based HIIT trial, by determining; 1, suitability and feasibility of eligibility criteria, 2, numbers of eligible participants from the target population, 3, willingness of PwP to be randomized, 4, baseline factors most strongly associated with the identified primary outcome to inform potential stratification in a full trial, 5, recruitment/retention rates and suitability of procedures, 6, sample size calculation required for a fully powered RCT, and 7, resources required for a full trial.

## 2 Methods and Materials

The HH4P trial was undertaken in accordance with the previously described protocol [11], summarized below.

Reporting of the HH4P trial followed guidelines presented in the CONSORT 2010 statement: extension to randomized pilot and feasibility trials [12], and the CONSORT checklist for reporting of patient‐reported outcomes [13], (see supporting materials).

### 2.1 Registration

This trial was registered at clinicaltrials.gov, identification NCT05485428.

### 2.2 Trial Design/Roles and Responsibilities/Setting

HH4P was a parallel group, randomized, controlled feasibility trial, with mechanistic, physiological and clinical sub‐components. The Chief Investigator (CI; author C.H.) had overall responsibility for the implementation of the trial and was supported by three members of a supervisory team (ST; authors L.C., H.G., J.M.). The trial was overseen by an independent trial steering committee (TSC), consisting of one service user and a practicing clinician, who both provided written consent. All assessments took place in the Nutrition, Exercise and Health laboratory, and the Exercise and Physiology laboratory, University of Plymouth (UoP), UK. All exercise sessions were undertaken in participant homes. Data analysis was undertaken at the UoP and at Manchester Metropolitan University (MMU), UK.

### 2.3 Participant Eligibility and Recruitment Strategy

This trial recruited through two NHS sites in the Southwest of England, Parkinson's UK and through UoP social media. Upon emailing an expression of interest to the CI, potential participants were referred to an online PIS, including consent to undergo telephone screening, where the following eligibility criteria were applied; Inclusion criteria were, 1. Diagnosed with Parkinson's disease, 2. Aged 18 years or older (No upper limit), 3. Hoehn and Yahr [14] stages 1–3 (Mild to moderate disease severity, physically independent) [3], 4. Capacity to consent under the Mental Capacity Act 2005 [15], and sufficient cognitive ability to follow an exercise program, 5. Based at home with enough space to perform the HH4P exercise program [10]. 6. Willing and able to travel to assessment visits to UoP, 7. Access to a computer, Smart Phone, or tablet and to the internet. Exclusion criteria were; 1. The presence of a concurrent neurological condition, 2. Co‐morbidities that would prevent/be exacerbated by high‐intensity exercise, 3. Advised to not participate following medical consultation, 4. Participation in a contemporaneous clinical trial.

### 2.4 Feasibility Trial Procedures

**Figure 1:**
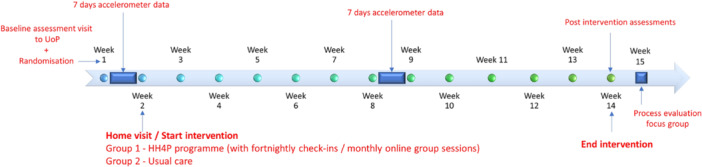
Schematic overview of HH4P feasibility trial procedures.

A schematic overview of trial procedures can be seen in Figure 1. Once eligibility was established, participants were invited to attend an initial assessment visit to UoP, where they completed a written consent form, bespoke health screening questionnaire [16], and all baseline assessments. On completion, an ActivPAL 3 (PAL technologies Ltd, Glasgow, UK) accelerometer was fitted to participants, who then underwent seven continuous days of physical activity (PA) data collection. Following the first assessment session, participants were randomized by minimization to either the HH4P HIIT program or usual care control. Minimization included a 1:1 ratio, and group stratification by sex and disease severity (Hoehn and Yahr stage 3/stage 2.5 or below). Randomization was undertaken with “Minim” MS‐DOS minimization software (University of York), by a blinded member of the ST. After 7 days postassessment, the CI undertook participant home visits (exercise and control) advising of group allocation, and to provide resources and ensure suitability of the exercise environment if applicable. During this time accelerometers were collected from participants. Participants then undertook 12 weeks of the HH4P HIIT program or usual clinical care. The HIIT group received fortnightly online Zoom (Zoom Communications inc. San Jose, California, USA) or telephone “check‐ins” throughout the program, were invited to attend monthly online group sessions and could receive SMS exercise reminders if required. Participants were also provided with Polar (Polar, Kempele, Finland) H9 heart rate (HR) monitors with data uploaded to Polar flow online (https://flow.polar.com) via the Polar flow smartphone application, and daily diaries to complete aspects such as exercise choice, duration and adverse effects and events (AE). Accelerometers were sent to all participants during week seven of the intervention to record a further 7 days of PA, then sent back to the CI in a prepaid envelope. Following the 12‐week program, all participants attended a follow‐up assessment visit at least 24 h after the final bout of exercise [11]. Exercise participants were also invited to attend a post intervention online focus group as a qualitative addition to process evaluation [17].

### 2.5 Interventions

Full description of the HH4P HIIT intervention and co‐creative development phase has been previously described in detail [10]. HH4P was a 12‐week, three times weekly home‐based HIIT program for PwP. HIIT sessions comprised of four sets of three 45 s bouts of high‐intensity calisthenic style exercises using minimal equipment, each bout interspersed with 15 s of rest, with a 2‐min rest period following each set [3, 10]. Initial target exercise intensity was 75% HR_max_ [10, 18], which was to be titrated up accordingly throughout the program to account for physiological adaptation, following evaluation of HR data and consultation between the CI and participant. HH4P included a number of exercise options, adaptations, and sequences to facilitate disease stage, ability and preference, with appropriate physical and online resources. These included online exercise instructional videos, and three stylistic variations of an original, musical rhythmic auditory cueing (RAC) accompaniment with interval timings and prerecorded verbal instructions [10].

Participants allocated to the control group continued to receive usual clinical care, which included continuing with any medications and PA routines, or attending medical appointments. HIIT participants also received usual clinical care.

### 2.6 Outcomes and Assessments

Trial outcomes, measures, related objectives and evaluation time points are summarized in Table 1.

**Table 1:** Summary of trial outcome sets, measures, related objectives, and time points.

Outcome set	Outcome measure	Related aim/objective	Evaluation time point(s)
Baseline characteristics	Demographic:		
	Sex/age/ethnicity	B1, C4	Week 1
	Home circumstance/employment status	B1, C4	Weeks 1 and 14
	Anthropometric:		
	Body mass/height (BMI)	B1, C4	Weeks 1 and 14
	Lifestyle:		
	Smoking status	B1, C4	Weeks 1 and 14
	Diagnostic:	
	Hoehn and Yahr stage/Comorbidities/blood pressure	B1, C4	Week 1
		B1, C4	Week 1 and 14
	Current medications	B1, C4	Weeks 1 and 14
Proposed primary outcomes	Blood serum brain‐derived neurotrophic factor	B1, B2, C4	Weeks 1 and 14 (blinded assessor)
	Maximal oxygen uptake (VO_2max_)	B1, B2, C4	Weeks 1 and 14 (Incremental exercise test with blinded assessor)
	Unified Parkinson's Disease rating scale part III	B1, B2, C4	Weeks 1 and 14 (blinded assessor)
Proposed secondary outcomes	30 s sit to stand	B1, B2, C4	Weeks 1 and 14 (blinded assessor)
	Oxford participation and activities questionnaire acute (Divided into 3 domains; 1. Routine activities, 2. Emotional wellbeing, 3. Social engagement)	B1, B2, C4	Weeks 1 and 14 (blinded assessor)
	Maximum heart rate	A4	Week 1 (during incremental exercise testing)
	Physical activity levels	B1, B2	Week 1 and week 7 (participant, 7‐day monitoring with accelerometers)
Feasibility: Adherence and engagement	Attendance at online group sessions	A2	Exercise participants only, 3 × during program
	Frequency and duration of exercise sessions	A2	Continual, participant recorded in diaries
	Web‐based heart rate monitoring (Polar Flow)	A2	Continual monitoring by the CI
	Program completion	A3	Week 14
Feasibility: Process effectiveness	Achieved heart rate	A4	Continual, self‐recorded with Polar HR monitor/Polar Flow online
	Rate of perceived exertion	A4	Continual, self‐recorded in diaries
Safety	Adverse/serious adverse effects & events	A1	Continual, self‐recorded, reported to the CI in the case of a serious adverse event
Participant acceptability: Qualitative evaluation	Participant focus group	A5, A6, C3	All exercise participants (Week 15 post randomization)
Process evaluation	Intervention fidelity	A7	Continual participant diary and feasibility data/Week 15 post randomization
	Total resource	C7	Week 15 post randomization
Eligibility, recruitment, and retention	Expressions of interest/numbers screened/numbers recruited	C1, C2, C5, C6	During recruitment phase/continual

Abbreviations: CI, chief investigator; HR, heart rate.

All trial outcomes were discussed and selected within the program co‐creation process [10]. Assessments were undertaken in accordance with official protocols as previously outlined [11] by a blinded member of the ST fully trained in each protocol, and who was a qualified phlebotomist with over 10 years of experience. Physical activity was monitored through accelerometery in both weeks one and seven, to measure and compare habitual PA throughout the program for both trial arms. Post intervention focus groups were undertaken with Zoom online platform, administered by the CI and one other member of the research team. Focus groups were based on a pre‐determined question checklist (see supporting material) relating to the acceptance of the exercise intervention and delivery procedures, along with aspects of the trial (such as outcome measures).

### 2.7 Sample Size

As a feasibility trial, an a‐priori power calculation was not appropriate [19]. Therefore this trial aimed to recruit 24 participants to enable evaluation of feasibility and safety factors, along with estimates of variability to inform sample size calculations for a full trial [20].

### 2.8 Data Analysis

Data analysis included the completion of CONSORT diagrams detailing aspects of recruitment and participation, baseline data summary and analysis, analysis of feasibility and safety outcomes, and production of descriptive statistics of proposed primary and secondary outcomes. Interval estimates of the potential pooled effects relative to usual care in the form of a 95% confidence interval were produced [19], to ensure that the effect size chosen for powering a future trial was plausible. Intention to treat (ITT) analysis was undertaken to analyse participants based on the groups they were randomized to regardless of treatment received, with any missing data imputed using a measure of central tendency. Outliers were defined as ±three SD from the mean. Normality of data was established by using the Shapiro‐Wilk test and through histogram evaluation, with pooled pre and postgroup results presented as either mean (SD), or median (IQR), depending on distribution. Between group differences were presented as mean (95% CI) or median (95% CI) accordingly, with 95% CI for the difference between median values calculated using the Hodges‐Lehmann estimation method. Quantitative data were analysed with Microsoft Excel version 2204 (Microsoft Corporation, Redmond, Washington, USA), and IBM SPSS, version 27 (IBM, Armonk, New York, USA). Serum BDNF was analysed at MMU by a researcher blinded to group allocation, with the BDNF sandwich enzyme linked immunosorbent assay kit (St. John's Laboratory, London, UK). The post intervention focus group was transcribed verbatim, and analysed with thematic analysis per protocol [11, 21], using NVIVO qualitative data analysis software (QSR International, Southport, UK).

### 2.9 Ethical Approval

Ethical approval for this trial was granted by the Health Research Authority (HRA; reference [22]/ES/0018) and UoP Faculty of Health Research Ethics and Integrity Committee (FHREIC; reference 3644). Recruitment via non‐NHS channels commenced on the August 23, 2023, and via NHS recruitment centers on the September 22, 2023.

## 3 Results

### 3.1 Recruitment and Participant Flow

**Figure 2:**
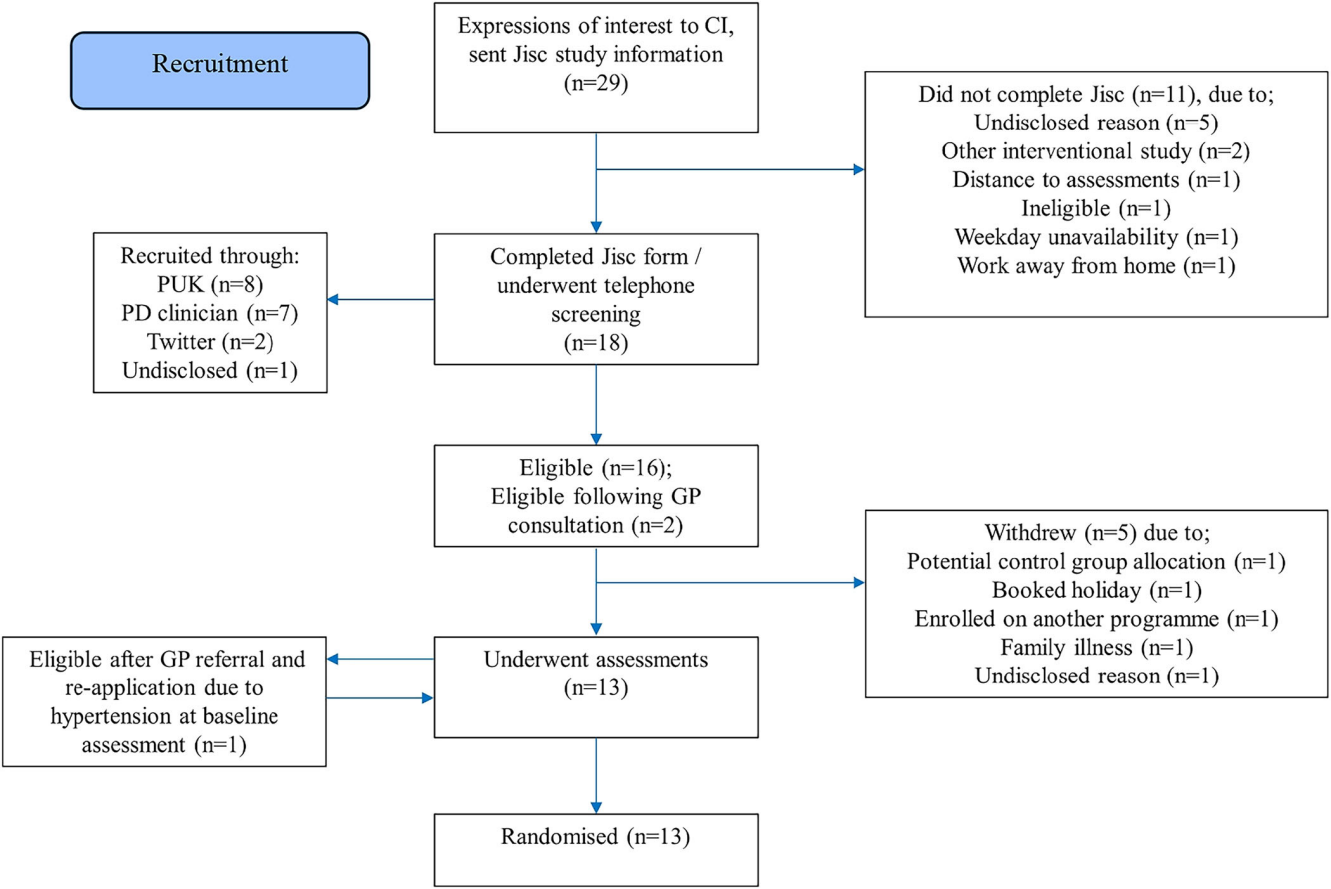
Recruitment flow CONSORT diagram.

**Figure 3:**
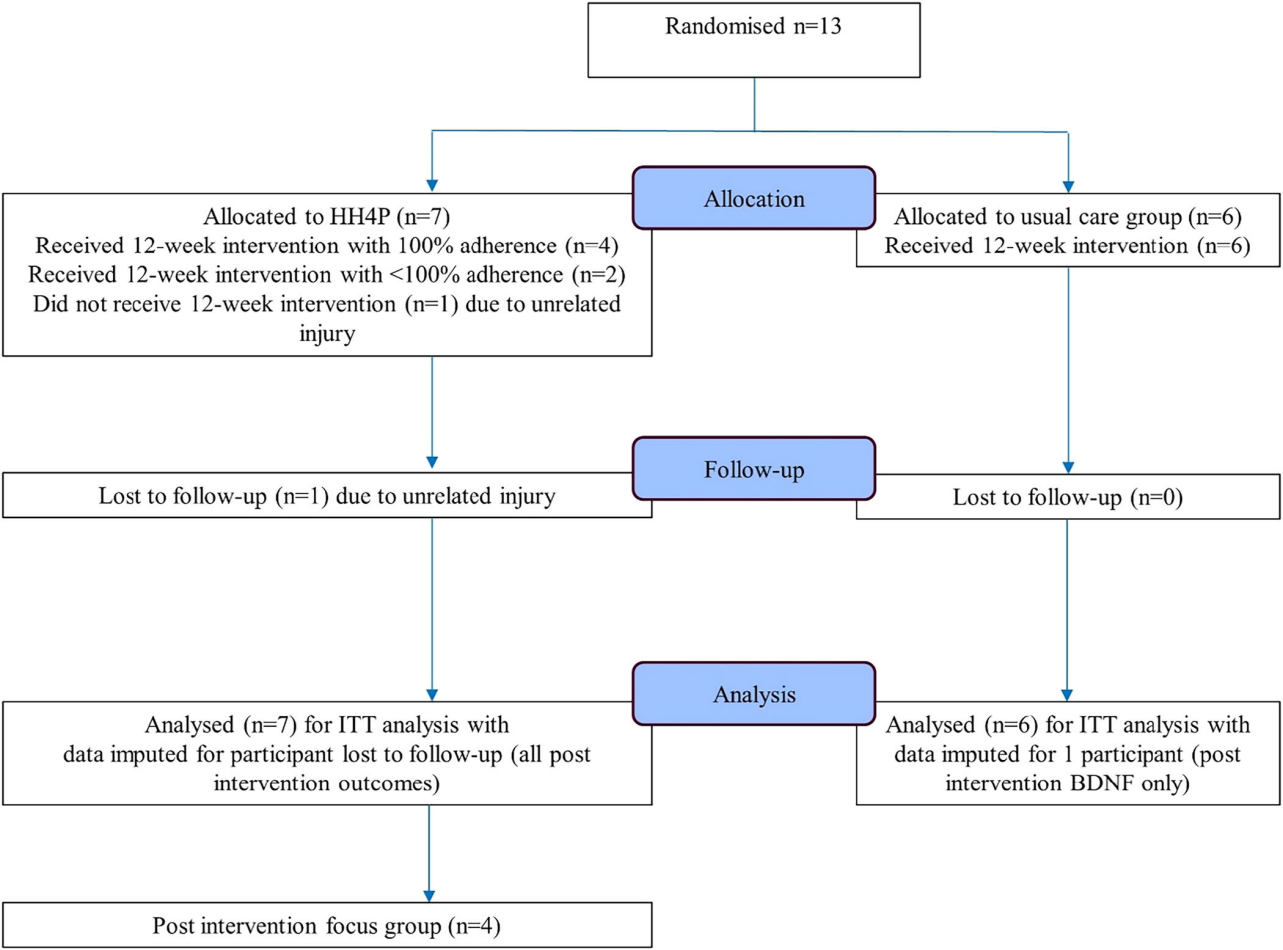
Participant flow CONSORT diagram.

CONSORT diagrams of recruitment and participant flow can be seen in Figures 2 and 3.

Twenty‐nine expressions of interest were received, of which 62% (*n* = 18) completed online questionnaires and consented to telephone screening. Of these, 89% (*n* = 16) were eligible, with the remaining (*n* = 2) deemed eligible following GP consultation. Following telephone screening, five people withdrew before undertaking assessments. In total, thirteen participants were recruited over a period of 3 months and completed baseline assessments. Random allocation per protocol allocated seven participants to the HIIT group, and six to the control group. Six HIIT participants (85.7%) completed the program and follow‐up assessments, with one unable to complete the program or assessments due to an unrelated back injury. Of the control group, all six participants attended the program and follow‐up assessments. Four exercise participants took part in the post intervention focus group.

### 3.2 Baseline Data; Anthropometric, Diagnostic and Demographic Characteristics

Baseline data were collected for all 13 participants, and can be seen in Table 2.

**Table 2:** Anthropometric, demographic and diagnostic baseline participant characteristics.

Group	Anthropometric			Diagnostic			Demographic		
	Age (Years)	Sex	BMI	H and Y stage	Time since diagnosis (years)	Medication	Home circumsta‐nce	Smoker	Employment
**HIIT (*n*** = **7)** (Mean {SD} for continuous data)	61.8 ( ± 9.4)	4 M/3 F	23.6 ( ± 1.8)	1.4 ( ± 0.8)	3.1 ( ± 3.1)	6 LD 1 DA	Six living with spouse/one living alone	None	3 F/T, 2 P/T, onenone, one retired
**Usual care (*n*** = **6)** (Mean {SD} for continuous data)	65.2 ( ± 2.3)	4 M/2 F	26.2 ( ± 3.0)	1.8 ( ± 1.0)	4.3 ( ± 3.1)	5 LD 1 DA	Five living with spouse/one living alone	None	1 P/T, five retired

Abbreviations: BMI, body mass index; DA, dopamine agonist; F, female; F/T, full‐time; H and Y, Hoehn and Yahr; LD, Levodopa; M, male; P/T, part‐time; SD, standard deviation.

### 3.3 Changes in Baseline Characteristics Postintervention

During the course of the program, four participants reported changes in medication dose (one HIIT participant and three control group). All other baseline data were comparable post intervention, with the exception of minor changes in weight equating to nonclinically significant changes in BMI, all being < 2 points [23].

### 3.4 Trial Steering Committee Meetings

The TSC met three times throughout the course of the program, at 3 months, 6 months and a concluding meeting at 10 months. No AE or serious AE requiring discussion were reported at any time.

### 3.5 Aim A. To Evaluate the Feasibility, Safety and Acceptability of a 12‐Week Home‐Based HIIT Program for PWP

A HIIT group summary of feasibility and safety outcome results can be seen in Table 3.

**Table 3:** Summary of HIIT feasibility and safety outcomes.

	Feasibility/safety outcome	Total
HIIT group completion and adherence	Program completion	85.7% (6/7)
	Adherence (Mean [SD], range)	78.4% (±29.9, 25–100)
	Sessions completed in full	100%
	Attendance at online group sessions	40%
	Completed “check‐ins”	76%
Process effectiveness; work phase exercise intensity	Number of sessions with objectively recorded HR	185/198
	% of HIIT sets achieving mean 75% HRmax	65.3
	Mean (SD) HR per session (% of HRmax)	77.2 (±6.1)
	% of participants achieving mean overall 75% HRmax	57.1
	Number of sessions with participant recorded RPE	171/198
	Mean (SD) RPE per session	15.1 (±1.8)
HIIT safety	HIIT related AE	Mild knee/ankle pain
		Knee pain
		Mild shoulder pain
		Mild calf/foot pain
		General fatigue
	HIIT related serious AE	None

Abbreviations: AE, adverse effect/event; HIIT, high‐intensity interval training; HR, heart rate; HR_max_, maximum heart rate; RPE, rate of perceived exertion; SD, standard deviation.

### 3.6 Program Safety

Five separate exercise participants reported minor AE related to the exercise in diaries and one in fortnightly check‐ins. These however were ameliorated by participants adapting the exercise sequence independently, or following consultation with the CI during fortnightly check‐ins. No further exercise related AE were experienced by the participants who went on to complete the program in full. Adverse effects that were reported by exercise participants to be unrelated to the exercise program included one lower back injury, rendering the participant unable to exercise for 4 weeks of the program, and consequently withdrew at week 11. Other unrelated AE included gastrointestinal and respiratory illness, and Achilles tendonitis. Whilst reported as unrelated to the intervention, these aspects were reported as negatively impacting the participants' engagement in the exercise program. The control group reported two occurrences of respiratory illness and one occurrence of newly diagnosed osteoarthritis.

### 3.7 Program Completion and Adherence

Six out of seven participants completed the full 12‐week HH4P program. All participants in the control group completed the 12 weeks of usual care. In total, HIIT participants completed 198 exercise sessions (792 sets). Four participants achieved 100% adherence (36/36 sessions), while the remaining participants achieved 69%, 55% and 25%. The overall group mean (SD) exercise adherence was 78.4% ( ± 29.9).

### 3.8 Online Group Sessions

In total, three optional online group sessions were undertaken. The first, with the option to undertake exercise, was attended by one out of six people invited. The second and third, which consisted of social interaction only were attended by five out of seven invited, and two out of seven respectively. Overall, this equated to group session attendance of 40%.

### 3.9 Participant Check‐Ins and Exercise Reminder SMS

In total, 33 out of the anticipated 42 (76%) HIIT participant check‐ins were undertaken, with 31 by telephone and two online. Two participants also requested SMS exercise reminders, and received these for the entire 12‐week program.

### 3.10 Achieved Exercise Intensity

As a measure of process effectiveness, HR data for 185/198 undertaken exercise sessions was successfully recorded by HR monitors, and the rate of perceived exertion (RPE, 6 to 20 Borg scale) [22] was recorded for 171/198 sessions in participant diaries. On two separate occasions, two participants achieved a higher HR than during the baseline incremental exercise test (IET) maximal HR assessments. Consequently, target intensity and achieved intensity results to date were amended accordingly. Overall, mean target exercise intensity (75% HR_max_) was achieved in 65.3% of sets, with a mean (SD) work phase intensity per session of 77.2% (±6.1) HR_max._ Individually, three participants did not achieve overall mean target intensity. The mean participant RPE was 15.1 (±1.8) per session. On one occasion, one participant achieved a noticeable ongoing increase in exercise intensity following a check‐in with the CI through modification of the program. Target intensity adjustments and consequent program modifications such as modifying shoulder exercises were suggested to four other participants, but did not result in a noticeable change in HR or RPE.

### 3.11 Participant Acceptability of the HH4P Exercise Program and Delivery Procedures

Four exercise participants attended the online post‐intervention focus group which lasted for 45 min. Three participants attended for the entire duration, while the fourth attended for the final 15 min only. Following deductive thematic analysis, four main themes were identified; “motivating factors,” “acceptable program procedures,” “demotivating factors,” and “unacceptable program procedures,” with subthemes identified relating to each. Participants also identified potential refinements to both the exercise intervention and delivery procedures. Table 4 shows identified themes and sub‐themes.

**Table 4:** Post intervention focus group themes and subthemes.

Main theme	Subthemes
Motivating factors	Being part of a group; Continual monitoring by researchers; Inter/intra participant competition through access to anonymised online participant data; Being proactive; Contemporary relevance of HIIT; Program inclusivity.
Acceptable program procedures	Online and physical resources; HIIT protocol (exercise type/timings etc); Exercise adaptations; Exercise and sequence variety.
Demotivating factors	Lack of social interaction opportunities; Potential isolation.
Unacceptable program procedures	Musical backing style; Shoulder exercises perceived to be ineffectual at stimulating appropriate intensity
Potential refinements	Increased number of whole group and subgroup social interaction opportunities; Adoption of a peer support model of supervision; Replacing the shoulder exercise set with a potentially higher intensity alternative; Blinding participants to pre and post outcome assessment scores; Provision of a wider range of musical styles to increase acceptability of RAC.

Abbreviations: HIIT, high‐intensity interval training; RAC, rhythmic auditory cueing.

### 3.12 Practicality of Resources and Intervention Fidelity

Self‐reported data from participant diaries (type and frequency of exercise and resources used) indicated that a range of exercises and resources were correctly and safely utilized in accordance with the protocol in 100% of undertaken sessions. Participants also appeared to have been able to adopt adaptations to exercise and sequence as demonstrated in the supporting resources to minimize AE and to maximize accessibility. While these factors, along with adherence and focus group data generally indicate a high degree of concordance between the intervention protocol and delivery, due to lack of achieved exercise intensity, the program in terms of overall exercise dose was not delivered as planned for some participants.

### 3.13 Aim A: Discussion and Preliminary Recommendations

Aim A of this feasibility trial was to evaluate the feasibility, safety and acceptability of the HH4P program. The occurrence of only minor, transient exercise related AE's, 85.7% exercise participant completion rates, and 78.4% adherence indicate that the HH4P program could be safe and feasible ‐ findings similar to previous HIIT programs for PwP [3]. Additionally, qualitative data suggests that the HH4P program and delivery procedures were acceptable to participants, and achievable to undertake, with only minor refinements suggested. However, three out of seven participants did not achieve mean initial target intensity, and the overall pooled mean intensity of 77.2% HR_max_ was close to the defined lower limit of “high intensity” exercise [18].

The lack of exercise related AE or serious AE indicate the 12‐week HH4P program to be safe for PwP of mild to moderate disease severity. However, the long‐term safety of HIIT for this population is still unknown. In agreement with previous evidence [3, 4], PwP of Hoehn and Yahr stages 1–3 appear to be willing and able to complete various modalities of HIIT style exercises safely over a 12‐week period. Encouragingly, it appears that a home‐based, calisthenic style program with appropriate resources and remote supervision, individualized adaptations and differentiation as advised by Gallo [24] can also be safely undertaken by PwP. However, this trial does not provide evidence regarding the safety of generic, nonindividualised HIIT programs for PwP in the home setting. HIIT therefore, should not be considered safe to be undertaken in the home without the appropriate consideration of individualization and methods of remote support. Additionally, the HH4P feasibility trial does not provide evidence to suggest that HIIT is a safe exercise option for people of higher disease severity. A previous systematic review [3] concluded that there was currently insufficient evidence to suggest that HIIT was safe for people of Hoehn and Yahr stages four to five. Further research is therefore required to explore the potential for increased intensity exercise options for this sub‐section of the population.

Completion of and adherence to the 12‐week HH4P program, along with qualitative data indicated the program to be both feasible and acceptable to participants. One key finding from post intervention focus groups, was the positive motivation experienced by participants due to online HR monitoring and fortnightly check‐ins. Although supervision for HH4P was remote, participants expressed a desire to adhere to the program due to being continually monitored by the CI. This indicates that ongoing monitoring, be it in person or remote, could be important for continued exercise adherence. As found by Paul et al. [7] and Ellis et al. [6] participant supervision has been identified by PwP as an important factor not just for safety, but adherence to exercise for PwP. This factor has obvious implications for participation and sustainability of home‐based exercise undertaken within a larger trial with reduced clinician support, or outside the parameters of a clinical trial. One potential avenue for further exploration in this regard is the application of peer support, as highlighted in the post intervention focus group. Peer support constitutes support from people with similar conditions or experiences, including one‐to‐one mentoring [25]. Peer support during home‐based exercise interventions for PwP could be an alternative option to clinician supervision to potentially maintain long‐term adherence. Further evaluation is required to explore the use of either online or in‐person peer support, possibly compared to conventional and/or remote clinician supervision to assess the influence on home‐based exercise motivation, adherence and benefits for PwP. The use of peer support could also provide the element of social interaction and feelings of being part of a group, outlined by participants as important for motivation and adherence, and is in agreement with previous research [6]. Access to online HR data through the “Polar Flow” internet site appears to have been a motivating factor for participants, adding an element of intra and inter participant challenge and competition. This form of peer interaction may be useful for some participants, including those not wishing to engage with in‐person or online social opportunities. As found by Cooke et al. [26] adding a competitive element to an aerobic exercise task could increase effort, enjoyment, HR and overall performance through various physiological and psychological mechanisms. Therefore, further evaluation of the use of competitive strategies could be considered for home‐based exercise programs such as HH4P. With regard to intensity, three participants were unable to achieve initial target intensity, with one‐third of all undertaken HIIT sets not achieving 75% HR_max_. Additionally, HR data suggested that potential adaptations were not sufficient to raise exercise intensity as participants progressed through the program. It is unclear however, if this was a result of the exercises and resources being ineffectual, or if participants were not able or willing to increase intensity, possibly due to factors such as form, or lack of motivation and self‐efficacy. Previous similar HIIT programs for PwP [4, 27], demonstrated the ability for participants to achieve a higher mean HIIT intensity, although these were all supervised programs. This factor could have improved intensity through in person encouragement [28], observation (Hawthorne effect) [29], and increased confidence and tuition leading to improved execution [30]. Congruently, the mean HIIT work phase HR for the exercise participants within the supervised development stage of this study, was 3.6% HR_max_ higher (80.8% compared to 77.2%), than participants undertaking the remotely supervised home‐based feasibility trial. Evaluations of the influence of differing supervisory models on exercise intensity, including home exercise clinician supervision would appear to be of importance. Exercise supervision comparisons should also include financial cost evaluations, to explore the practicality and cost effectiveness of differing supervisory models. The lack of achieved intensity in HH4P could also have been a result of the calisthenic style exercises, compared to the equipment‐based exercise, such as the use of cycle ergometers, or “Speedflex” resistance machines, used in previous literature [3, 4]. Whilst importantly, participants were able to modify HH4P exercises to maximize safety and completion, the failure of exercises and adaptations to increase intensity leads to uncertainty regarding the appropriateness of calisthenic style, home‐based exercises for the use of HIIT. This highlights the requirement for future studies to evaluate the differences in achieved intensity stimulated by differing HIIT modalities such as resistance machines, compared to the HH4P calisthenic style exercise protocol. Such studies could also evaluate the merits of various adaptations for each exercise modality. Also, whilst use of RAC was deemed acceptable by participants, the efficacy of RAC accompaniments for maintaining movement amplitude and frequency remains uncertain, and requires further investigation. As a matter of course, all future piloting involving home‐based HIIT that explores avenues to increase exercise intensity, should also include well considered, contemporaneous safety and feasibility assessments to ensure appropriateness for the Parkinson's population. Overall, similar to previous HIIT programs for PwP, the 12‐week, home‐based HH4P program appears to be safe and acceptable for PwP of mild to moderate disease severity. This indicates that with suitable resources, adaptations and supervision, home based HIIT can be as safe as supervised HIIT within clinical settings, and could provide a suitable short‐term exercise option. However, whether calisthenic style exercises utilized in HH4P are suitable to generate the required intensity remains in doubt, while the most appropriate form of supervision and model of support require further exploration. Therefore, until further research as outlined is undertaken, whether the HH4P program could be a safe, feasible, long‐term high‐intensity exercise option for PwP remains uncertain.

### 3.14 Aim B. To Identify the Clinical and Physiological Outcomes That Could be Feasible and Sensitive to Change Compared to Usual Care in a Full Home‐Based HIIT Trial for PWP

#### 3.14.1 Potential Primary and Secondary Outcome Measure Feasibility and Completion (Table 5)

**Table 5:** Completeness of potential primary and secondary outcome measures.

Potential primary/secondary outcome	Timepoint	Completeness	
		% participants undertaking outcome	% of potential data collected
BDNF	Pre	100	92
	Post	92	85
VO_2max/_HR_max_	Pre	100	100
	Post	92	92
MDS‐UPDRS III	Pre	100	100
	Post	92	92
30 s STS	Pre	100	100
	Post	92	92
OxPAQ acute	Pre	100	100
	Post	92	92
Physical activity	Pre	100	62
	Week 7	100	15
Total (mean)	Pre	100	92
	Post/Week 7	93	78

Abbreviations: 30 s STS, thirty second sit to stand; BDNF, brain derived neurotrophic factor; HR_max_, maximum heart rate; MDS‐UPDRS, Movement Disorder Society unified Parkinson's Disease rating scale; VO_2max_, maximal oxygen uptake.

##### 3.14.1.1 Blood Sampling and BDNF Analysis

Blood samples were obtained per protocol for all but two assessment attendees. Only preintervention samples were obtained from one participant, as difficulties were experienced with blood extraction during postintervention assessments. Also, while both pre and post intervention samples were obtained from another participant, the pre intervention sample was not processed in duplicate due to complications within the centrifuge process. Other than this, samples were successfully processed and stored in a −80° freezer pending analysis. In total, 24 microtubes of blood serum were successfully transported to MMU, with BDNF data obtained and analysed for both time points for all samples.

##### 3.14.1.2 VO_2max_/HR_max_ ‐ Incremental Exercise Tests

In total, 25 IET's were performed per protocol [11], with 24 terminated due to volitional exhaustion, and one due to a plateaux in VO_2_ and HR. The mean (SD) IET length was 11 m 44 s (± 2 m 40 s; range 7 m 0 s to 15 m 4 s), with a mean RER of 1.23 ( ± 0.06; range 1.11–1.33) and mean RPE of 17.3 ( ± 1.44; hard; range 14–20). Both RER and RPE results substantiated the achieving of maximal exercise capacity. Consequently, all tests were considered as “good tests” deemed to have achieved maximal exercise capacity, and all IET VO_2max_ and HR_max_ data were included within analysis. All IET's were completed with no AE.

##### 3.14.1.3 MDS‐Updrs III/Oxpaq acute/30 STS

In total, 25 assessments for the MDS‐UPDRS, OxPAQ acute and 30 STS were completed. The MDS‐UPDRS demonstrated a floor effect, (a score of 0 indicating no noticeable symptoms) whereupon several scores were too low to be sensitive to improvement. The OxPAQ acute appeared to be a time efficient assessment of participation in daily activities, with an approximate completion time of two to 3 min. As with the MDS‐UPDRS, a strong floor effect was evident with seven participants scoring 0 points at pre‐intervention assessments for at least one of the three OxPAQ acute domains, thereby limiting potential improvements, and resulting in skewed data. Thirty second STS tests were completed by participants according to the official protocol with no AEs.

##### 3.14.1.4 ActivPAL Accelerometer Data Collection

Seven days of habitual PA was measured objectively through accelerometery for a period of 1 week at baseline and in week seven of the HH4P program, for all participants in both exercise and control groups. No accelerometers were removed prematurely, and no concerns such as skin irritation were highlighted. Following the baseline week all accelerometers were retrieved during home visits. However, following week seven, two monitors were lost whilst in transit back to UoP. Following data checking, 8/13 datasets covering the baseline week, and 2/13 (both intervention group participants) covering week seven were deemed suitable for analysis.

##### 3.14.1.5 Baseline and Week Seven PA Data

Analysis of five datasets revealed the exercise group to have undertaken a mean 77,662 steps (range 30,716–108,556) with a mean estimated energy expenditure (EES) of 245.1 met hours (met.h; range 224.9 to 257.9) during the baseline week. This equated to an average of 11,095 steps/35.0 met.h EES per day. Comparatively, analysis of three control group datasets revealed a mean 38,358 (range 29,730–55,544) steps during this time period, with a mean EES of 228.3 met hours (223.2–234.2), or 5480 steps/32.6 met.h per day. The exercise group also completed a greater mean amount of standing time, stepping time, sitting/lying time, upright to seated lying events and less sitting/lying time than the control group. Due to missing data for week seven, group comparisons of PA undertaken during baseline week and week seven were not possible.

In total, the percentage of potential data collected in pre intervention assessments was 92%, and 78% in postintervention assessments.

##### 3.14.1.6 Responsiveness to Change in Mechanistic, Physiological and Clinical Outcomes

Table 6 shows a summary of pre and post results for proposed primary and secondary outcomes, difference between groups (HIIT ‐ control), and minimum clinically important difference where applicable. Reduction signifies improvement for the MDS‐UPDRS III and all domains of the OXPAQ acute.

**Table 6:** Summary of results for each outcome.

Primary/secondary outcome (Mean {SD} unless stated)	Minimum clinically important difference	HIIT group (*n* = 7**)			Usual care group (*n* = 6***)			Between group difference, HIIT ‐ control: Mean, (95% CI)
		Pre	Post	Change	Pre	Post	Change	
Brain‐derived neurotrophic factor (pg/mL)	N/A	2344 (±947)	1663 (±1028)	−681 (±1126)	2823 (±1433)	1792 (±1050)	−1031 (±910)	350 (−915 to 1615)
VO_2max_ (mL/Kg/min)	+/−2	29.0 (±5.7)	29.9 (±5.4)	0.9 (±1.7)	24.5 (±5.3)	24.0 (±5.7)	−0.5 (±2.0)	1.4 (−0.9 to 3.7)
MDS‐UPDRS III^1^	+/−5 points	6.9 (±4.1)	5.7 (±5.4)	−1.2 (±4.8)	7.0 (±3.6)	6.5 (±3.3)	−0.5 (±3.9)	−0.7 (−6.3 to 4.9)
30 s STS (number of stands)	N/A*	15.6 (±1.6)	20.1 (±3.6)	4.5 (±2.4)	15.3 (±2.3)	14.8 (±2.1)	−0.5 (2.1)	5.0 (2.2 to 7.8)
OxPAQ acute^1^								Median (95% CI)
Routine activities (Median {IQR})	+/−7.5	11.0 (2.0 to 24.0)	7.0 (2.0 to 16.0)	−4.0 (−8.0 to 0.0)	10.0 (1.8 to 16.8)	8.0 (1.8 to 24.8)	0.0 (−6.8 to 0.0)	−4.0 (−9 to 12)
Emotional wellbeing (Median {IQR})	+/−10.8	35 (12.5 to 42.5)	20 (7.5 to 35.0)	−5 (−17.5 to 0.0)	12.5 (2.5 to 37.5)	5.0 (1.3 to 5)	−7.5 (−17.5 to −1.3)	2.5 (−20 to 15)
Social engagement (Median {IQR})	+/−5.5	0.0 (0.0 to 15.5)	0.0 (0.0 to 6.5)	0.0 (−12.5 to 0.0)	9.0 (0.0 to 18.8)	3.0 (0.0 to 15.8)	0.0 (−5.3 to 0.0)	0.0 (−13 to 19)

Abbreviations: CI, confidence intervals; HIIT, high‐intensity interval training; IQR, inter‐quartile range; MDS‐UPDRS, Movement Disorders Society unified Parkinson's disease rating scale; mL/Kg/min, milliliters per kilogram per minute; OxPAQ, Oxford participation and activities questionnaire; pg/mL, picograms per milliliter; SD, standard deviation; VO_2max_, maximal oxygen uptake.

* No suggested parameters for PwP.

** Imputed data for 1 participant for all post outcomes.

*** Imputed data for 1 participant post BDNF only.

^1^ Reduction signifies improvement.

Based on predefined criteria, no outliers were identified that warranted removal from any data set.

Of the potential primary outcomes following the 12‐week program, the HIIT group experienced an increase in VO_2max_ of 0.9 ( ± 1.7) mL/Kg/min, (equating to a 3% increase), a 29% reduction in BDNF (−681 {±1126}) pg/ml and a −1.2 ( ± 4.8) point reduction in the MDS‐UPDRS III. Of the potential secondary outcomes, the HIIT group increased in the 30 s STS, (4.5 ( ± 2.4) stands equating to a 22.4% increase), and decreased in the routine activities and emotional wellbeing subsections of the OxPAQ acute (median {IQR} −4 points {−8.0 to 0.0} and −5 points {−17.5 to 0}) respectively. The only potential primary or secondary outcome to improve in the HIIT group compared to the control, was the 30 s STS with a between group difference of mean (95% CI) 5.0 stands (2.2–7.8).

### 3.15 Aim B: Discussion and Preliminary Recommendations

Aim B of this feasibility trial was to identify the clinical and physiological outcomes that could be feasible and sensitive to change compared to usual care. Overall, preliminary data indicated that outcomes were feasible and practical to administer, but only the 30 s STS appeared to be sensitive to change compared to usual care following 12 weeks of home‐based HIIT.

Whilst high levels of completion and data collection indicated the majority of potential primary and secondary outcomes to be feasible, the most obvious practical limitation regarding the HH4P delivery was the remote use of accelerometers. Participants informally commented that placing accelerometers correctly at the week seven timepoint without the help of a researcher was problematic. Inadequate fitting guidance could have led to incorrect leg placement during the seventh week of monitoring, in turn causing the monitor to fail to recognize the distinction between lying, and sitting/standing events [31]. Therefore, future studies requiring remote data collection, should ensure adequate participant training either in person or remotely, and consider the potential limitations of postal procedures when formulating data collection protocols. In contrast to previous, nonhome‐based HIIT studies for PwP, the results of potential primary outcomes in HH4P failed to indicate the benefits of HIIT compared to usual care. This factor raises several uncertainties regarding the HH4P program and delivery procedures. The lack of clinically important improvement in VO_2max_ could be explained when compared to healthy controls, by factors such as comparatively reduced maximal cardiac output due to dysfunction of the sympathetic division of the autonomic nervous system [32]. However, findings in this study are in contrast to a similar, nonhome based, controlled feasibility trial with PwP [4], who used a comparable HIIT protocol with regard to timings and overall exercise volume, with a similar population (Hoehn and Yahr stages 1–3). Harvey et al. [4] demonstrated HIIT group improvements of 2.8 mL/Kg/min, compared to 0.9 mL/Kg/min in this trial. Therefore, differing factors such as achieved intensity or exercise modality may have been influential. The mean intensity in Harvey et al. [4] was 85% HR_max_, while in this feasibility trial it was 77.2% HR_max_. Similarly, Duplea [27] found VO_2max_ improvements of 4.7 mL/Kg/min in a HIIT group who achieved a mean exercise intensity of 92% HR_max_. It is a possibility therefore, that the reduced intensity achieved in HH4P could have resulted in the relative lack of overall benefit to VO_2max_. This would appear to be supported by Schenkman et al. [33] who found that high‐intensity treadmill exercise (80%–85% HR_max_) was more beneficial to cardiorespiratory fitness than moderate intensity continuous exercise (MICE; 60%–65% HR_max_) in newly diagnosed PwP. It should be noted however, that the 8% improvement from baseline experienced by the high intensity participants in Schenkman et al. [33] was following a 6‐month training period, twice the length of HH4P. It is a possibility therefore, that if the HH4P program was of a similar duration, greater benefits to cardiorespiratory fitness may have been found. Although, Atakan et al. [34] found that five HIIT sessions over 1 week stimulated similar improvements in VO_2max_, as the same amount of sessions of similar intensity over a 2 week period in healthy males. Results were attributed to possible skeletal muscle mitochondrial adaptations acutely increasing maximum arteriovenous oxygen difference [35]. This finding indicates that HIIT session frequency, rather than overall program duration may be important for VO_2max_ improvements. Consequently, further evaluation exploring the influence on VO_2max_ of differing exercise frequencies for the HH4P program is advised, alongside appropriate feasibility outcomes. Additionally, the generally raised baseline fitness of participants in HH4P HIIT group compared to the control could have attenuated between group differences in VO_2max_. The HIIT group were assessed as having considerably higher VO_2max_ levels at baseline than the control group (29.0 mL/Kg/min compared to 24.5 mL/Kg/min), which was also higher than the estimated mean aerobic capacity of 22.2 mL/Kg/min of the general Parkinson's population [36]. Also, the higher level of baseline fitness in the exercise group appears to be substantiated by reduced BMI and higher levels of baseline PA (possibly influenced by employment status) [37]. Group stratification therefore, by cardiorespiratory fitness or baseline PA levels may be justified within future studies and within further evaluations of the HH4P program. The lack of apparent benefit to VO_2max_ following HIIT could also have been influenced by session exercise volume within the HH4P program. With 9 min of high‐intensity exercise included per session, the HH4P program could be described as “low volume” HIIT, as in, HIIT that includes less than 15 min of high‐intensity exercise [38]. Although, as the HH4P HIIT session volume was broadly based on previous HIIT routines for PwP [4] that resulted in an improvement in VO_2max_, exercise volume may not have been the main influence regarding the indicated lack of positive change in HH4P. Additionally, evidence now suggests that low volume HIIT is at least as beneficial as high volume HIIT for a number of cardiometabolic health factors in various populations [38]. However, future comparisons of the HH4P protocol with differing exercise session volumes and work/interval timings is recommended.

The indicated lack of benefit of HIIT to serum BDNF appears to be congruent with VO_2max_ results. A recent systematic review including 16 exercise studies and 370 PwP [39], found that exercise intensity had a positive linear association with BDNF, possibly due to factors such as hyperthermia resulting in increased BBB permeability [40], brain hypoxia [41], and increased circulation of lactate molecules [42]. Therefore, the lack of exercise intensity achieved in this trial may also have been influential to the apparent absence of change in BDNF following HIIT. In agreement, MDS‐UPDRS III motor symptom assessments in this trial did not indicate mean “on phase” benefits, in contrast to previous studies of HIIT with PwP [27, 43], although no increase in symptoms was demonstrated. Findings in both groups were similar, and demonstrated considerable between‐subject variability, leading to greater uncertainty of these results. The lack of indicated benefits in the HIIT group could be explained by factors such as exercise intensity or absence of improvement in BDNF [44]. Also, a psychometric limitation of the MDS‐UPDRS III that was noticeable within the current trial, was a floor effect (zero being no noticeable symptoms) limiting the potential to identify motor symptom improvements in early Parkinson's [45]. Therefore, for people with early stage Parkinson's, the MDS‐UPDRS III may not be apposite for motor symptom assessment. As with the MDS‐UPDRS III, the OxPAQ also demonstrated a floor effect, with participants scoring zero on baseline assessments for various subsections on 14 occasions. As such, the lack of positive change in either group was not unexpected. The relatively early stage Parkinson's of HH4P participants, could have influenced the low scoring rendering the OxPAQ acute insensitive to improvements. Further HH4P evaluations should also consider alternatives to the OxPAQ acute for assessing changes in ADL in PwP of mild to moderate disease severity. Of the proposed primary and secondary outcomes, the 30 s STS appeared to be the only outcome sensitive to change compared to usual care following 12 weeks of HIIT. Findings agree with a review by Tillman et al. [46] who found that eight to 24 weeks of lower‐body resistance training significantly improved lower‐limb strength in PwP of mild to moderate severity. As with HH4P, several of the studies included in the review such as Allen et al. [47] utilized body‐weight resistance training, and achieved similar benefits to machine‐based programs. The HH4P 30 s STS results also support the principle of specificity. The HH4P program included sit to stand/squat exercises, similar to the 30 s STS, whilst previous studies have shown that HIIT cycle ergometer training can significantly improve cycling endurance [48]. Further studies should therefore evaluate various forms of HIIT training specific to patient‐centric goals.

Overall, preliminary data suggests that the majority of primary and secondary outcomes undertaken within the HH4P feasibility trial appear to be feasible, and acceptable to participants, although alternatives to the MDS‐UPDRS III and the OxPAQ acute should be considered for this population. However, the indicated lack of change in potential primary outcomes, possibly due to limitations within the HH4P exercise intervention and delivery procedures, prohibit the identification of a primary outcome for a full trial.

### 3.16 Aim C: To Elucidate the Key Methodological Considerations for the Implementation of a Full Home‐Based HIIT Trial

Aim C of this feasibility trial was to elucidate the key methodological considerations for a potential full trial, such as sample size and suitability of eligibility criteria. Whilst a number of methodological factors regarding the implementation of a full trial could be tentatively discussed, full consideration of these factors is only appropriate when a suitable rationale for progression can be presented. Despite high levels of program engagement and outcome completion, due to the lack of improvements in potential primary outcomes, a full HH4P trial cannot be recommended until further evaluations as previously discussed indicate that progression may be appropriate.

#### 3.16.1 Feasibility Trial Limitations

The limited sample size of this feasibility trial is an important consideration ‐ 13 participants over a 3 month period were recruited. This constituted just over half of the target sample size, and indicates that recruitment strategies did not fully reach the intended audience. Due to timescale limitations, the recruitment period was not extended. Consequently, the recommended feasibility trial sample of 24 as discussed by Julious et al. [20] was not achieved, limiting the certainty of conclusions. Similarly, data saturation in the focus group was unlikely due to the lack of attendees. Also, as a feasibility trial, several pertinent outcomes found to be improved by previous HIIT programs for PwP [3], such as endothelial reactivity and anti‐inflammatory myokine interleukin‐6 were not explored. Additionally, as a purely self‐reported outcome open to forms of bias such as social desirability [49], the use of the OxPAQ acute could be debated.

## 4 Conclusion

This feasibility trial aimed to assess the feasibility, safety and acceptability of a co‐created home‐based HIIT program for PwP, and elucidate potential primary and secondary outcomes, along with key methodological considerations for a full trial. Overall, results indicate the program to be safe, acceptable to participants, and feasible to undertake for PwP of mild to moderate disease severity. However, whilst the administration of primary and secondary outcomes appears feasible, the lack of potential benefits experienced by participants, along with the inability to achieve greater exercise intensity raises uncertainties regarding the HH4P intervention and methodology. Therefore, factors such as exercise type, frequency, model of support, outcome measures, and sample size require further evaluation before progression to a full trial can be considered. Addressing these uncertainties could provide a suitable rationale for a definitive home‐based HIIT trial with long‐term follow‐up. In turn, this could further elucidate the potential of this exercise modality as an appropriate, extended exercise strategy for PwP.

## Discrepancy From Study Protocol

Following author consideration, objective Biii as published within the trial protocol [11] was omitted from this article due to perceived lack of relevance.

## Author Contributions

**Conrad Harpham:** conceptualization, investigation, writing – original draft, methodology, writing – review and editing, software, formal analysis, project administration, resources, visualization, data curation, validation, funding acquisition. **Hilary Gunn:** conceptualization, investigation, writing – review and editing, methodology, supervision, software, project administration, resources, validation, funding acquisition. **Jonathan Marsden:** conceptualization, writing – review and editing, software, supervision, project administration, methodology, resources, validation, funding acquisition. **Raul Bescos‐Garcia:** investigation, writing – review and editing, methodology, supervision, project administration, resources. **Christopher Murgatroyd:** investigation, writing – review and editing, methodology, software, formal analysis, data curation, resources, validation. **Luke Connolly:** conceptualization, investigation, writing – review and editing, methodology, project administration, software, supervision, data curation, resources, validation, funding acquisition.

## Conflicts of Interest

The authors declare no conflicts of interest.

## Transparency Statement

The lead author Conrad Harpham affirms that this manuscript is an honest, accurate, and transparent account of the study being reported; that no important aspects of the study have been omitted; and that any discrepancies from the study as planned (and, if relevant, registered) have been explained.

## Supporting information

Additional supplementary materials HSR.

Supplementary materials HSR.

## Data Availability

The data that support the findings of this study are available from the corresponding author upon reasonable request.
