# Supplementary material for: Home‐Based High‐Intensity Interval Training for People With Parkinson's: A Randomized, Controlled, Feasibility Trial
Source: Health Sci Rep. 2025 Jul 14;8(7):e71024. doi: 10.1002/hsr2.71024 (PMC12257130; doi:10.1002/hsr2.71024)
Supplement: Supplementary file 1 — Additional supplementary materials HSR. [file HSR2-8-e71024-s002.docx]

**HIIT-Home4Parkinson’s post intervention focus group agenda**

Purpose: To explore the acceptance of the HH4P exercise programme and delivery procedures, practicality of intervention resources and willingness of participants to be randomised. Any possible adaptations will also be identified.

- Location: Online (Zoom).
- Number of groups: 1
- Participants per group: 6 people with Parkinson’s
- Duration: 45 minutes.
- Facilitator: Conrad Harpham (Chief Investigator), plus one supervisor.

**General meeting requirements**

- Access to Zoom
- Secure Wi-Fi connection
- Quiet room with no interruptions
- Blurred background

**Schedule**

- Introductions, ongoing consent and confidentiality (ground rules).
- Exercise participants will initially be asked to talk individually about their experience.

Specific prompts relating to the HIIT exercises will include;

- Aspects of the programme and delivery procedures deemed to work well
- Aspects of the programme and delivery procedures requiring development, and how to adapt the programme accordingly
- Acceptability / practicality of physical and online resources
- Suitability of the programme for the home environment
- Thoughts regarding randomisation
- Motivational aspects (group sessions, online researcher check-ins)
- Participant acceptability of measures / assessments
- Track questions to completion and follow-up on themes.
- Conclude: Acknowledge and thank participants. Discuss plans for dissemination
- Data will be recorded, transcribed verbatim and analysed with thematic analysis techniques
